# Eta polycaprolactone (ε-PCL) implants appear to cause a partial differentiation of breast cancer lung metastasis in a murine model

**DOI:** 10.1186/s12885-023-10813-6

**Published:** 2023-04-13

**Authors:** Benjamin Benzon, Sandra Marijan, Matij Pervan, Vedrana Čikeš Čulić

**Affiliations:** 1 Department of Anatomy, Histology and Embryology, University of Split, School of Medicine, Split, Croatia; 2 Department of Medical Chemistry and Biochemistry, University of Split, School of Medicine, Split, Croatia; 3 Medical Studies Program, University of Split, School of Medicine, Split, Croatia

**Keywords:** ε-PCL implants, Murine breast cancer, Differentiational therapy of cancer

## Abstract

**Background:**

Cells in every epithelium can be roughly divided in three compartments: stem cell (SC) compartment, transient amplifying cell (TA) compartment and terminally differentiated (TD) compartment. Maturation of stem cells is characterized by epithelial stromal interaction and sequential maturational movement of stem cell’s progeny through those compartments. In this work we hypothesize that providing an artificial stroma, which murine breast cancer metastatic cells can infiltrate, will induce their differentiation.

**Methods:**

BALB/c female mice were injected with 10^6^ isogenic 4T1 breast cancer cells labeled with GFP. After 20 days primary tumors were removed, and artificial ε-PCL implants were implanted on the contralateral side. After 10 more days mice were sacrificed and implants along with lung tissue were harvested. Mice were divided in four groups: tumor removal with sham implantation surgery (*n* = 5), tumor removal with ε-PCL implant (*n* = 5), tumor removal with VEGF enriched ε-PCL implant (*n* = 7) and mice without tumor with VEGF enriched ε-PCL implant (*n* = 3). Differentiational status of GFP + cells was assessed by Ki67 and activated caspase 3 expression, thus dividing the population in SC like cells (Ki67^+/dim^ aCasp3^−^), TA like cells (Ki67^+/dim^ aCasp3^+/dim^) and TD like cells (Ki67^−^ aCasp3^+/dim^) on flow cytometry.

**Results:**

Lung metastatic load was reduced by 33% in mice with simple ε-PCL implant when compared to tumor bearing group with no implant. Mice with VEGF enriched implants had 108% increase in lung metastatic load in comparison to tumor bearing mice with no implants. Likewise, amount of GFP + cells was higher in simple ε-PCL implant in comparison to VEGF enriched implants. Differentiation-wise, process of metastasizing to lungs reduces the average fraction of SC like cells when compared to primary tumor. This effect is made more uniform by both kinds of ε-PCL implants. The opposite process is mirrored in TA like cells compartment when it comes to averages. Effects of both types of implants on TD like cells were negligible. Furthermore, if gene expression signatures that mimic tissue compartments are analyzed in human breast cancer metastases, it turns out that TA signature is associated with increased survival probability*.*

**Conclusion:**

ε-PCL implants without VEGF can reduce metastatic loads in lungs, after primary tumor removal. Both types of implants cause lung metastasis differentiation by shifting cancer cells from SC to TA compartment, leaving the TD compartment unaffected.

**Supplementary Information:**

The online version contains supplementary material available at 10.1186/s12885-023-10813-6.

## Introduction

Every epithelial tissue is in a constant process of turnover [1, 2]. Regardless of complexity, cells in each epithelium can be roughly divided in three compartments: stem cell (SC) compartment, transient amplifying cell (TA) compartment and mature, terminally differentiated (TD) or functional cell compartment [3]. Furthermore, it seems that stem cell compartment is composed of at least two different subpopulations [3, 4]. A quiescent one, that is more likely to survive noxious influences and subsequently be the basis of tissue regeneration; and an active stem cell subpopulation that constantly proliferates and thus serves as basis of constant epithelium renewal. Differentiation of stem cells to mature epithelial cells is characterized by sequential maturational movement of its progeny through all three compartments [3]. This sequence usually ends with apoptosis or detachment from stroma into lumen followed by apoptosis (i.e. anoikis) [2, 5]. Moreover, apoptosis seems to be a differentiation taken to extreme when it comes to a morphological features and molecular machinery [6, 7]. A significant body of evidence suggests that underlying stroma with its morphogenetic gradients of various physical (e.g. voltage, mechanical force) and chemical (e.g. bone morphogenic protein, Wnt, Hedgehog (HH)) factors is instrumental in a such process of differentiation [8, 9].

Over the last 70 years, a body of evidence that interruption of stromal – epithelial interactions can lead to the development of carcinoma has been accumulated and united to a certain extent under the theoretical framework know as Tissue Organization Field Theory (TOFT) of cancer [10]; a very thorough review is given in a recent article by Baker [11]. Here we will point out the three most striking studies. Back in 1951. Billingham et al. published a study in which cancers were induced chemically on the mice skin [12]. When chemically treated epidermis (i.e., epithelium) was transplanted on nontreated dermis (i.e., stroma) no cancers developed. On the other hand, when nontreated epidermis was transplanted to treated dermis in 62% of cases cancers developed. 22 years later Karp et al. showed that filters with pore size less than 22 μm, when implanted subcutaneously in mice, induce sarcomas; whereas filters with greater pore size did not cause neoplastic transformation [13]. In 2003. Maffini et al., similarly to Billingham, showed that chemical carcinogenesis in rat mammary gland is independent of epithelium and that stroma is the crucial target in process of carcinogenesis [14]. In the last two decades, cancer genome sequencing studies have revealed that most of the driver mutations involve genes and proteins that are part of morphogen signaling pathways (e.g. NOTCH, HH, APC/Wnt) [15]. When it comes to histological architecture, all of the three epithelial compartments can be found in tumor [16, 17]. The proportion of each compartment varies with the histological grade of neoplasia, with the stem cell compartment being the most abundant in poorly differentiated tumors [18].

**Fig. 1:**
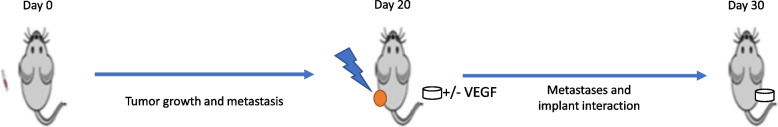
Experimental design. 14 wk. old BALB/c female mice were injected with 10^6^ isogenic 4T1 murine breast cancer cells labeled with GFP. After 20 days, mice were again anesthetized with isoflurane and primary tumors were excised en-block. Artificial ε-PCL implants were implanted subcutaneously on the contralateral side immediately after the tumor excision. After 10 more days mice were sacrificed and implants along with the lung tissue were harvested for flow cytometric analysis for quantification of metastatic burden and their differentiation state

Mathematical modeling of stem cell differentiation by maturational movement trough epithelial compartments points out that differentiating tumor stem cells might be more efficacious than killing them [19]. Furthermore, metastatic process is characterized by so called metastatic inefficiency which manifests as abundance of quiescent micro-metastases relative to proliferating metastases [20, 21]. Here, given the evidence from above, we speculate that purpose of metastatic process might be providing an additional stroma which might be able to differentiate or induce quiescence in incoming metastatic cells. Stroma is made of extracellular matrix and fibroblasts, in experiments presented here mice were treated with artificial extracellular matrix made of eta polycaprolactone (ε-PCL) in hope that this would provide additional niches for differentiation of metastatic cancer cells (Fig. 1). Moreover, we enriched some of the implants with vascular endothelial growth factor (VEGF) so that the link between the implants and blood circulation might be established earlier, which could at least theoretically accelerate infiltration of the implants by circulating tumor cells and thus hypothetically speed up the process of differentiation.

## Materials and methods

### Mice and experimental design

Fourteen wk. old BALB/c female mice were injected with 10^6^ isogenic 4T1 murine breast cancer cells labeled with green fluorescent protein (GFP, Bioware® Brite 4T1-Red-FLuc-GFP, Perkin Elmer, Boston, Massachusetts, USA) in right mammary line, under isoflurane anesthesia. Cells were grown in RPMI media with 10% FBS without antibiotics in atmosphere supplemented to 5% CO_2_ at 37 °C. After one or two passages they were considered to be fit for injection into mice. After 20 days, mice were again anesthetized with isoflurane and primary tumors were excised en-block. Furthermore, artificial ε-PCL implants (3D Biotek Polycaprolactone (PCL) scaffold inserts, fiber diameter ≈300 μm, ≈300 μm spacing, Merk KGaA, Darmstadt, Germany) were implanted subcutaneously on the contralateral side. One eight (1/8) of the original scaffold volume was implanted. After 10 more days mice were sacrificed and implants along with the lung tissue were harvested for flow cytometry. Based on intervention, mice were divided in four groups: tumor excision with sham implantation surgery (*n* = 5, control group), tumor excision with ε-PCL implant (*n* = 5), tumor excision with vascular endothelial growth factor (VEGF)—enriched ε-PCL implant (*n* = 7) and mice without tumor with VEGF—enriched ε-PCL implant (*n* = 3) (Fig. 1). This partial factorial design enables the study of effects of implants on tumor load in lung tissue as well as the effect of metastatic process and implants on differentiation of lung metastases. Some implants were enriched with VEGF so that a link between circulatory system and the implant might be established sooner or in more abundant manner [22], which could at least theoretically increase the number of metastases that migrate in to the implant.

All experimental procedures were approved by the Animal Welfare Committee, Directorate for Veterinary Affairs, Ministry of Agriculture, Republic of Croatia. The study is reported in accordance with ARRIVE guidelines.

### Coating of ε-PCL implants with heparin and VEGF

Ε-PLC scaffolds were coated with VEGF after Singh et al. [22]. In brief, ε-PLC implants were incubated in 0.05 M 2-(N-Morpholino)ethanesulfonic acid (MES) buffer (pH = 5.5) for 15 min. Next, the scaffolds were immersed in freshly prepared solution of heparin (1% w/v), 0.5 M N-(3-Dimethyla-minopropyl)-N0-ethylcarbodiimide hydrochloride (EDC) and 0.5 M N-hydroxysuccinimide (NHS) in MES buffer and stirred briefly. After 15 h of incubation at room temperature, the scaffolds were extensively washed with distilled water to remove the byproducts.

At this point scaffolds that would not be coated with VEGF were stored at 4 °C in 1 mL of PBS overnight. On the other hand, scaffolds that would be coated with VEGF were covered with 10 μg of recombinant mouse VEGF (164aa) from E. coli (Applied Biological Materials Inc., Richmond, British Columbia, Canada) dissolved in PBS and dried in laminar hood for 40 min. After this, the scaffolds were immersed in 1 ml of PBS at 4 °C for overnight before implantation to remove freely diffusible VEGF and to reduce the possible in vivo burst release of VEGF.

### Flow cytometry

Lungs and ε-PCL implants were explanted on day 30 of experiment during the necropsy, whereas primary tumor was removed on day 20 by the surgical en block excision. After that, the tissues were disintegrated mechanically by mincing and digested enzymatically for 1 h on 37 °C in 10 mg/mL Collagenase type I (Sigma Aldrich, Merck KGaA, Darmstadt, Germany) solution in RPMI, 5 ml of the solution was applied per 100 mg of tissue. Single cells solutions of tissues were fixed in 0.1% paraformaldehyde solution for 15 min followed by permeabilization in 0.1% solution of TritonX in PBS, also for 15 min. Next, cells were stained by two antibody panels. First one was composed of anti-GFP (clone B-2, Santa Cruz Biotechnology, Santa Cruz, California, USA), ant- Ki-67 (clone B56, BD Biosciences, Franklin Lakes, New Jersey, USA) and anti-activated caspase 3 (aCasp3, clone C92-605, BD Biosciences) antibodies. Second panel included PI (BD Biosciences) and anti GFP antibody (Santa Cruz Biotechnology). Samples were analyzed by Accuri C6 (BD Biosciences) flow cytometer. Gating controls were prepared with Fluorescence Minus One (FMO) stains of tumor cells and appropriate biological controls (i.e., samples from healthy mice). Three methods were used for drawing gates (i.e. discriminating positive and negative events): for FMO controls based gates 3% background method was used; for comparison of an experimental sample with biological control, percentile by percentile histogram subtraction method was used if cumulative probability curve of sample was uniformly shifted to the higher florescence level in comparison to biological control, if the latter was not the case, then the maximum difference method was used. The described gating methods were used either in one or two dimensions. Detail description of these methods can be found in paper by W. R. Overton [23] and textbook by Shapiro [24]. An illustration of gating plan is depicted in Figures S1 and S2. All data were analyzed with FCS Express software (De Novo Software, Pasadena, California, USA).

### Transcriptomics

Data for transcriptomic analysis were obtained from Gene Expression Omnibus (GEO) [25]. They were uploaded to Gene Pattern web suite [26], with GEOimporter tool, for further analysis. Furthermore, single sample gene set enrichment analysis with rank normalization was done with ssGSEA tool [27]; its output was further statistically analyzed as described in next subsection. Enrichment analysis of *MKI-67* and *Caspase 3* genes in certain gene ontology categories was done by PANTHER software with Fisher’s Exact Test without correction for multiple comparisons, on Gene Ontology website [28–30].

### Statistical analysis

Depending on distribution, continuous data are presented as either arithmetic mean or mode and range (range = max. – min.) or 0.5% trimmed range. These were estimated by fitting the normal, uniform, triangular, exponential, or power law distribution ($cdf={(\frac{x-\mu }{{x}_{max}-\mu })}^{a+1}, {x}_{max}>\mu , a>-1$). Relative changes in data are presented as ratios or relative differences (%) with control group values in denominator. Since interventions i.e., treatments or factors in experiment are ordered categories we chose modelling for trends (i.e., tests for trends) to be a mainstay of data analysis here. Data was modeled for trends with exponential, quadratic or linear function as deterministic part and Gaussian distribution as stochastic part of model; for model comparison purposes horizontal line was used as null model if not stated otherwise. Off note, these linear models are equivalent to t test when only 2 groups are considered. Furthermore, if data spread was of scientific interest, then the range or 0.5% trimmed range were modelled for trend. For nonparametric modelling the smoothening splines with 5 or 6 knots were used. Number of knots was based on the biology of the process that was modeled. Survival curves were calculated using the life table method and they were modeled by the exponential function. Details on models used in the paper are given in figure description or supplementary tables describing each dataset. As a measure of statistical evidence in favor or against hypotheses, R^2^ or η^2^, evidence ratio (ER) based on difference in Akaiake Information criterion (ΔAIC) [31] and *p* values were used. Standard errors (i.e., 68% confidence interval) were used as an uncertainty measure of the estimates, they were estimated by maximum likelihood or parametric (percentile) bootstrap method. When it comes to notation convention used throughout the paper, ER < 1 and ΔAIC < 0 favor the null model, a number following ± sign is the standard error, not the range nor any other descriptive statistic used to describe the spread of data (e.g., standard deviation). *P* values were calculated by extra sums of squares F test if not stated otherwise. They are interpreted according to ASA statement on *p* values [32]. Model diagnostics was done with residual plots, normality probability plots and Shapiro Wilk test where appropriate. Sample size was estimated by Mead’s resource Equation. [33].

## Results

### ε-PCL implants decrease metastatic load in lungs by approximately 30%

**Fig. 2:**
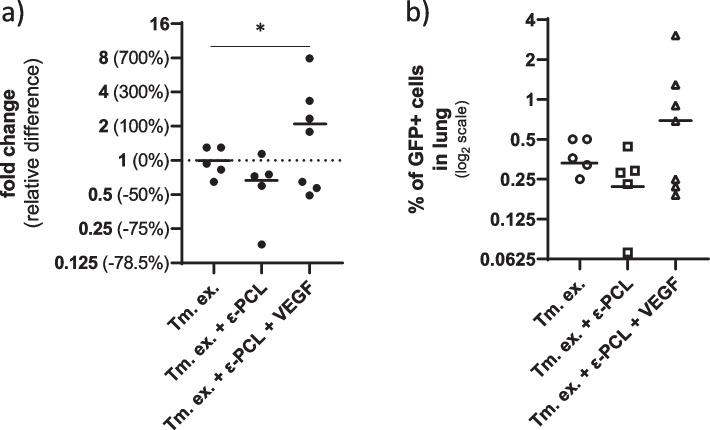
Effects on metastatic load in the lungs. **a** Relative changes in % of GFP + cells in lungs; a parabolic (quadratic) trend with the nadir at the group treated with excision of primary tumor and nonenriched ε-PCL implant (R^2^ = 71.13%, ΔAIC = 14.65, ER≈1.52·10^3^, p = 0.0002-*). **b** Proportion (%) of GFP + cells in lungs of mice on log_2_ scale; the Gaussian distribution was used to model data from groups that were treated with primary tumor excision only (μ = 0.33 ± 0.005, σ = 0.09 ± 0.01, R^2^ = 99%) and primary tumor excision with nonenriched implant (μ = 0.22 ± 0.02, σ = 0.13 ± 0.04, R^2^ = 90%), while the data from mice treated with excision of primary and VEGF enriched implant was modeled with exponential distribution (β = 0.7 ± 0.06, *R*^2^ = 94%). Legend: ε-PCL, eta polycaprolactone; μ, arithmetic mean; σ, standard deviation; β, scale parameter of the exponential distribution which equals arithmetic mean and standard deviation; standard error is used as a measure of uncertainty of an estimate

Implanting ε-PCL scaffolds with and without VEGF enrichment resulted in parabolic (quadratic) trend (R^2^ = 71.13%, ΔAIC = 14.65, ER≈1.52·10^3^, *p* = 0.0002) that can be described as follows: in relative difference terms ε-PCL implants decreased the lung metastatic load, on average, by 33 ± 6%; whereas, unexpectedly, enriching the ε-PCL implants with VEGF increased metastatic load by 108 ± 25%, when compared to control group (Fig. 2a). In absolute terms this means that ε-PCL implants decreased the metastatic load from 0.34% (range = 0.47%), found in control group, to 0.23% (range = 0.56%) of GFP + cells. On the other hand, enrichment of implants with VEGF increased the load to 0.70% (range: 3.72%) of GFP + cells (Fig. 2b). Furthermore, these data also point to a claim that, in addition to average increase in metastatic load, enriching the implants with VEGF increases heterogeneity (i.e., scatter) of response when compared to nonenriched implants (η^2^ = 80.05%, ΔAIC = 15.72, ER≈2.5·10^3^, *p* < 0.0001, t test).

### Tumor cells migrate from other metastatic sites to ε-PCL implants

**Fig. 3:**
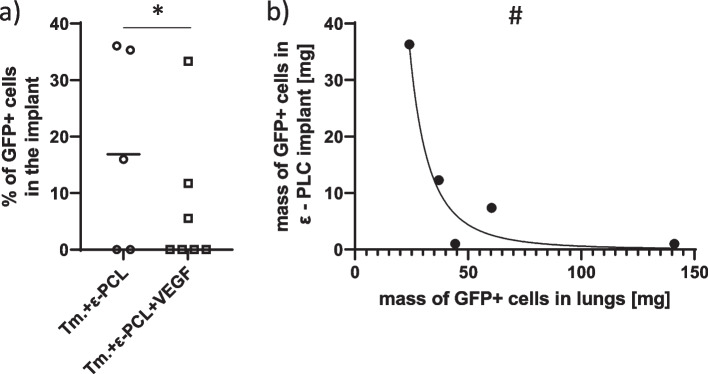
Infiltration of ε-PCL implants by the tumor cells. **a** Proportion (%) of GFP + cells in the implants; the Gaussian distribution was used to model data from both nonenriched (μ = 17.45 ± 5.1, σ = 16 ± 8.6, R^2^≈46%) and VEGF enriched (μ = 0 ± 0.5, σ = 10.5 ± 1.6, R^2^≈94%) implants. This formed a negative linear trend between implants (η^2^ = 73.1%, ΔAIC = 7.98, ER = 53.96, *p* = 0.0237, Welch’s t test- *). **b** A relationship of inverse proportionality between mass of metastatic (GFP +) cells in the lungs and mass of tumor (GFP +) cells that infiltrated the nonenriched implant ( $m\left(GFP+ cells\: in\: lungs\right)=b*{m\left(GFP+cells\: in\: the\: implant\right)}^{a}$, a = -0.34 ± 0.09, b = 144.711·10^3^, 68% CI (30·10^3^ to 462.063·10^3^), *R*^2^ = 94%, ΔAIC = 11.6, ER≈330, *p* = 0.007-#). Legend: ε-PCL, eta polycaprolactone; μ, arithmetic mean; σ, standard deviation; m (GFP + cell in …), a mass of GFP + cells in lungs or the implant measured in mg; standard error is used as a measure of uncertainty of an estimate if the interval estimate is symmetric, if it is asymmetric the 68% confidence interval is used

ε-PCL implants from both groups that received them were infiltrated with GFP + cells to the variable extent (Fig. 3a). 3/5 (60%) of ε-PCL implants that were not enriched with VEGF were infiltrated with tumor cells giving the average level of 17.7 ± 5% of GFP + cells. On the other hand, 3/7 (≈43%) ε-PCL implants enriched with VEGF were infiltrated with tumor cells giving the average level of 0.02 ± 1% of GFP + cells. Thus, the average effect of VEGF enrichment can be described as decrease in tumor cells infiltration by 17.45 ± 5.1% (η^2^ = 73.1%, ΔAIC = 7.98, ER = 53.96, *p* = 0.0237, Welch’s t test). There, also, seems to be influence of VEGF on heterogeneity of effect. Furthermore, when the relationship between metastatic loads in the lungs and ε-PCL implant without VEGF is considered, it turns out that those two are inversely proportional, i.e., increase in GFP + cells in the implants is associated with the decrease in lung metastatic load (*R*^2^ = 94%, ΔAIC = 11.6, ER≈330, *p* = 0.007) (Fig. 3b). Such relationship could not be found when data from mice treated with VEGF enriched ε-PCL implants was analyzed.

### Selection of a cell differentiation marker

During normal tissue turnover, mature cells end their maturational movement trough epithelial compartments and their lifecycle by apoptosis [1–3]. Due to its morphological and biochemical similarities with differentiation, apoptosis can be viewed as differentiation taken to extreme [6]. In last two decades, evidence of caspase 3 involvement in differentiation of numerous tissues that originate from mesoderm and ectoderm have been accumulated [34]. Moreover, it seems that caspase 3, and other caspases, should be considered a cell reshaping enzyme [34]; the difference between apoptotic and differentiational function of caspase 3 lies in its concentrational and temporal patterns of expression [6, 7].

**Fig. 4:**
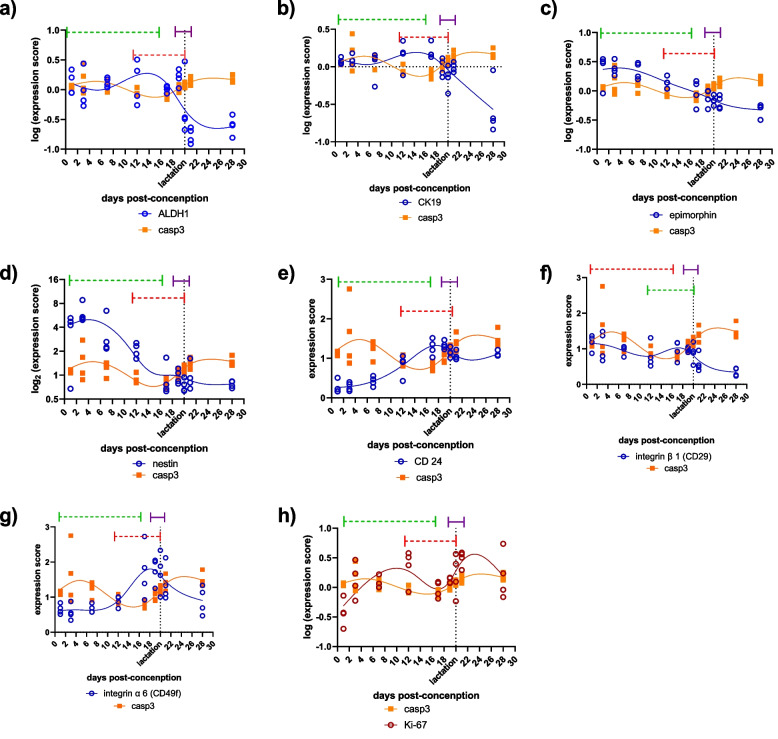
Time dynamics of activated caspase 3 mRNA and other markers of stemness during the breast development and differentiation in pregnant mice. Number of knots in cubic splines is equal to number of points that mark the beginning and the end of different stages in mouse mammary gland growth and differentiation during pregnancy, that is, 6 knots were used to fit the splines. Green dashed line marks the period of ductal morphogenesis and alveolar buds formation, red dashed line marks the period of secretory differentiation and purple line marks the period of peak differentiation

**Fig. 5:**
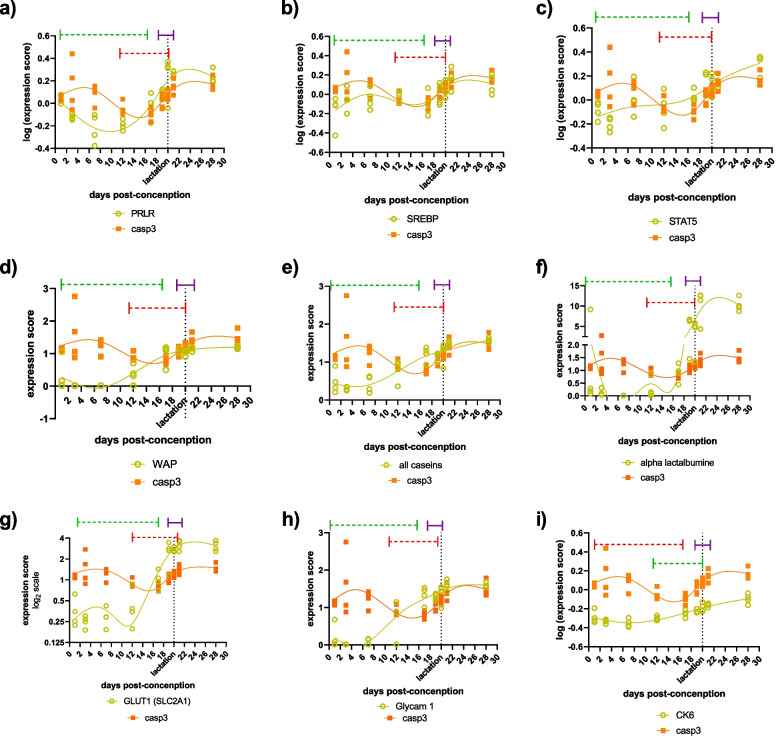
Time dynamics of activated caspase 3 mRNA and other markers of functional (i.e., fully differentiated) mammary gland during the breast development and differentiation in pregnant mice. Number of knots in cubic splines is equal to number of points that mark the beginning and the end of different stages in mouse mammary gland growth and differentiation during pregnancy, that is, 6 knots were used to fit the splines. Green dashed line marks the period of ductal morphogenesis and alveolar buds formation, red dashed line marks the period of secretory differentiation and purple line marks the period of peak differentiation

When it comes to breast tissue differentiation during pregnancy and lactation, here, we reanalyzed publicly available transcriptomic data from Lemay et al. [35] (GEO accession No.: GSE8191) in order to examen expression patterns of caspase 3 and markers of both stem and mature mammary cells (Fig. 4) [36–39]. Based on histological, biochemical and gene expression features, murine mammary tissue growth and differentiation during pregnancy and lactation is divided in three somewhat overlapping phases: ducal morphogenesis and alveolar bud formation, secretory differentiation, and peak differentiation [35, 40]. Casp3 follows temporal dynamics which is opposite to the dynamics of stemness markers (i.e., *ALDH1, CK19, CD24, CD29, CD49f*) in all phases of mammary growth and differentiation (Fig. 4 a, b, e—g). Furthermore, when it comes to temporal relationship with epimorphin, a morphogen that drives ductal growth and branching [41] and nestin, Casp3 again follows the opposite dynamic at the peak of differentiation (Fig. 4c, d). On the other hand, in secretory and peak differentiation phases caspase 3 follows the same time trends as prolactin receptor (*PRLR*) expression (Fig. 5a). Two key transcriptional factors which are responsible for synthesis of milk proteins (*STAT5*) and milk lipids (*SREBP*) also follow the same temporal expression pattern as caspase 3, during the differentiation phases (Fig. 5b, c). Moreover, the same can be noticed for marker of myoepithelial differentiation *CK6*, milk proteins (whey acid protein, caseins and α lactalbumin), milk mucins (*Glycam 1*) and glucose transporter (*GLUT 1*), which is involved in lactose synthesis. (Fig. 5d—i). Given what has been said in previous paragraph and these correlations in temporal patterns of expression (Table S1) we decided to use aCasp3 as a marker for cell differentiation. For descriptive purposes expression of Ki – 67 in breast differentiation is depicted in Fig. 4h.

### Differentiation of tumor cells in lung metastases

To study differentiation of tumor cells, based on cell turnover patterns in highly proliferative tissues such as hematopoietic tissue [42, 43] and findings exposed in previous paragraph, GFP + cells were divided into subpopulations that would mimic tissue compartments. Cells that were Ki-67^+/dim^ aCasp3^−^ were considered to be proliferating stem cell (pSC) like, those that were Ki67^+/dim^ aCasp3^+/dim^ were considered to be transient amplifying cell (TA) like. The nonproliferating subpopulation of GFP + cells (i.e., Ki-67^−^) was further divided into terminally differentiated or apoptotic like (TD) cells (Ki67^−^ aCasp3^+/dim^) and quiescent stem cell like (qSC) cells (Ki67^−^ Casp3^−^).

**Fig. 6:**
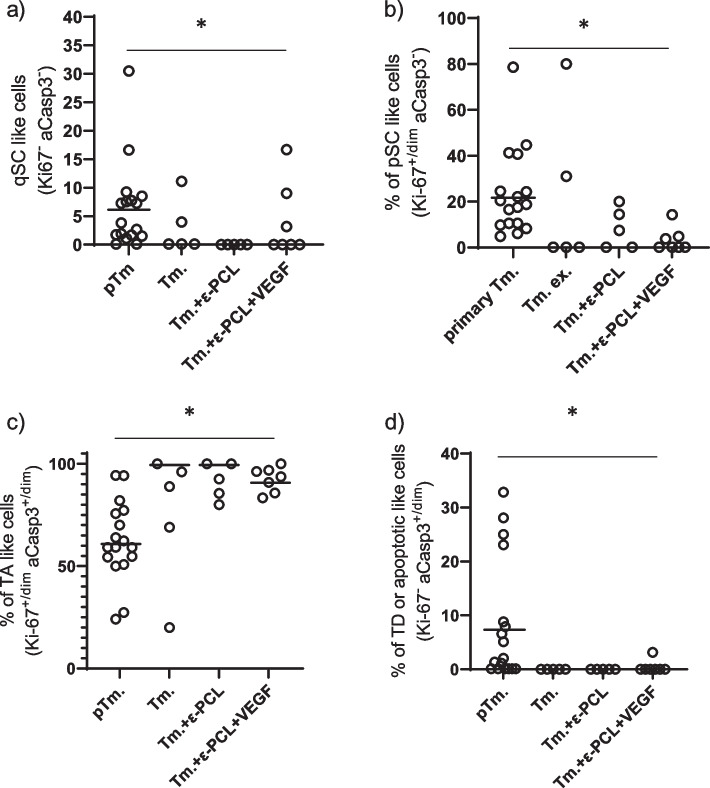
**a** Proportions (%) of quiescent stem cell (qSC) like cells among GFP + cells in lungs. When it comes to trends a decreasing exponential trend (R^2^ = 76.9%, ΔAIC = 44.75, ER > 10^4^, *p* < 0.0001) can be seen among averages. **b** Similar trend can also be noticed when it comes to proliferating stem cell (pSC) like cells. **c** Transient amplifying (TA) like cells show almost the mirror image of two previous trends, while again a decreasing exponential trend can be seen with terminally differentiated (TD) or apoptotic cells (**d**). More details on statistics can be seen in Tables S2 to S5. *—significant tests for trends described in text

In order to study the effects of both metastasizing and implants on various tissue compartments, primary tumor and lung metastases were compared (Fig. 6). The average fraction of quiescent cells (qSC, Ki67^−^ Casp3^+/dim^) in primary tumors was 5.64 ± 0.85%, whilest in metastases from mice that were only treated with primary tumor excision average was 0 ± 0.05%. Furthermore, mice treated with both kinds of ε-PCL implants also had an average of 0 ± ≈0%. This can be described as decreasing exponential trend (*R*^2^ = 57.9%, ΔAIC = 24.42, ER > 10^4^, *p* < 0.0001) with the average levels of qSC fraction being the same, i.e., 0% in all metastases regardless of treatment (Fig. 6a). When it comes to heterogeneity of the effects, a quadratic trend can be seen (*R*^2^ = 57.8%, ΔAIC≈26.5, ER > 10^4^, *p* < 0.0001), with the range of observed data being the greatest in primary tumors (32.4 ± 3.35%) and then decreasing in mice that had surgery only (11.2 ± 0.7%), with its minimum (0 ± ≈0%) in group that was treated with implantation of simple ε-PCL implant and then raising again in group treated with ε-PCL implant enriched with VEGF (17.54 ± 1.07%) (Fig. 6a).

Fraction of pSC like cells (Ki-67^+/dim^ aCasp3^−^) follows the same exponential trend as quiescent cells in terms of average response (*R*^2^ = 86.9%, ΔAIC = 64.2 ER > 10^4^, *p* < 0.0001) (Fig. 6b). That is, the primary tumors are characterized by the average fraction of 22 ± 1.5%, followed by the mice that were treated with tumor excision only (0 ± 0.34%), mice treated with the non-enriched implants (0 ± 0.37%) and mice treated with enriched implants (0 ± 0.23%). However, when it comes to heterogeneity of metastatic and treatment effects a negative linear trend can be seen (*R*^2^ = 91.38%, ΔAIC = 64.2 ER > 10^4^, *p* < 0.0001), with the range being the greatest in primary tumors (97.8 ± 0.01%) and surgery only group (80.58 ± 4.4%), decreasing to 20.7 ± 1.7% in mice treated with surgery and ε-PCL implant. Furthermore, the most homogeneous, i.e., the most uniform, effects were observed in mice treated with surgery and VEGF enriched implants; the range was 14.35 ± 0.54% (Fig. 6b).

The TA (Ki67^+/dim^ Casp 3^+/dim^) cell like fraction showed relationships that are the mirror image of those observed in pSC fraction (Fig. 6c). Primary tumors with the average of 60.7 ± 0.7% represent the group with the smallest fraction of TA like cells. Lung metastases from mice that were treated with surgery consisted, on average, almost entirely of TA like cells (100 ± ≈0%). Likewise, mice treated with surgery and ε-PCL implant wo. VEGF had 100 ± ≈0% of TA like cells in their lung metastases. Similarly, mice treated with surgery and ε-PCL implant enriched with VEGF had the average fraction of TA like cells of 91.26 ± 0.93%. These observations constitute a quadratic trend when it comes to the effects of metastatic process and treatments (*R*^2^ = 97.5%, ΔAIC = 119.8, ER > 10^4^, *p* < 0.0001). Influence of the factors on heterogeneity of response is similar to the one observed in pSC like cells.

Terminally differentiated or apoptotic (TD) like cells had the highest average fraction in primary tumors (6.07 ± 2.5%), followed by all other metastases regardless of treatment (≈0 ± 0%) (Fig. 6d). This can be described as descending exponential trend (*R*^2^ = 14.01%, ΔAIC = 3.898, ER = 5.58, *p* = 0.0292).

To summarize, the effects of metastatic process and implantation can be described as follows: metastatic process reduces the average fraction of pSC and qSC like cells when compared to primary tumor. The effect is made more uniform by both kinds of ε-PCL implants. The opposite process is observed in TA like cells compartment when it comes to averages. Finally, these changes in proportions of tissue compartments along with the compartments of healthy breast and lung tissue are depicted in Fig. S3 as ternary or compositional plot.

More details on statistical models are available in Table S2 to S5.

### Differentiation of tumor cells in ε-PCL implants

**Fig. 7:**
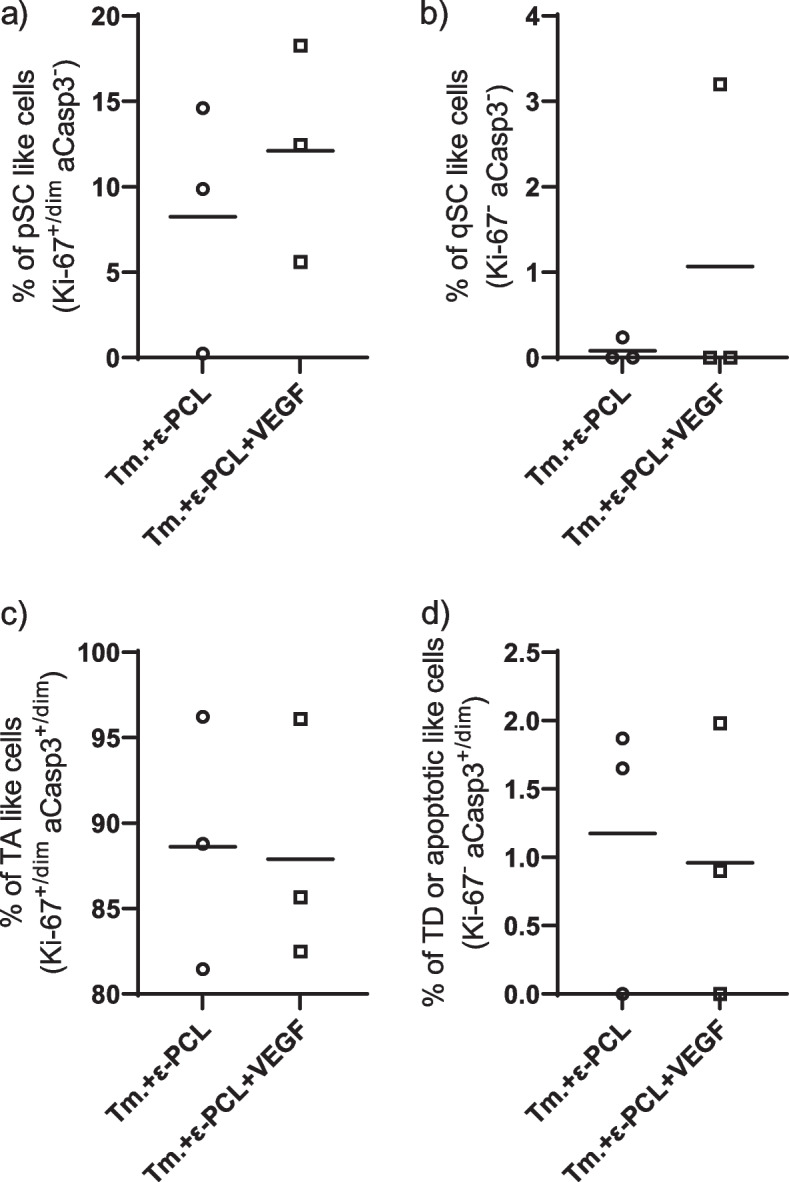
Proportions of different compartments among GFP + cells in the implants. Legend: qSC, quiescent stem cell; pSC, proliferating stem cell; TA, transient amplifying; TD terminally differentiated

Since only 3 implants were infiltrated by GFP + cells in each group treated by implantation ε-PCL scaffolds, this subsection will contain only non-inferential analysis (Fig. 7). When it comes to qSC like cells implants enriched with VEGF contained 1.1% (range = 3.2%) and non-enriched implants contained 0.08% (range = 0.24%) of such cells. pSC fraction is similar in both kinds of implants, with the average of 8.23% (range = 14.36%) in nonenriched and 12.1% (range = 12.65%) in VEGF enriched implant. Great majority of cells (88%) in both implants belongs to TA like cells. The amount of TD or apoptotic like cells is negligible in both types of implants.

### Gene expression signatures that mimic tissue compartments are associated with survival probability in metastatic breast cancer patients

To investigate what might be potential implications of changing the proportions of different tissue compartments in metastases we examined publicly available data from Brasó-Maristany et al. [44] (GEO accession No.: GSE175692). In this cohort 184 metastases were sampled and expression of 771 genes was measured. Since *Caspase 3* was not included in these 771 genes, we analyzed expression of gene sets related to differentiation and cell proliferation in which *Caspase 3* and *MKI- 67* were overrepresented. Trough the search of Gene Ontology Consortium database we identified two such GO terms: Cell fate commitment (enrichment score for *Caspase 3* was 84 and *p* = 0.03, with Fisher’s exact test) and Cell population proliferation for *MKI-67*. Based on GO terms two lists of genes associated with those terms in The Molecular Signatures Database were selected and their expression was analyzed by single sample GSEA. Based on ssGSEA score, the samples were divided by quartiles (Q1 – 4) of expression with the Q1 having the lowest and Q4 the highest expression. To mimic tissue compartments with genetic signatures samples were divided in groups that parallel those used on mice (Table 1).

**Table 1:** Construction of genetic signatures that mimic tissue compartments

**Tissue compartment signature**	Cell fate commitment** gene list expression quartile**	logical** connective**	Cell population proliferation** gene list expression quartile**
qSC like	Q1	and	Q1
pSC like	Q1	and	Q2 or Q3 or Q4
TA like	Q2 or Q3 or Q4	and	Q2 or Q3 or Q4
TD like	Q2 or Q3 or Q4	and	Q1

*Legend*: *qSC* quiescent stem cell, *pSC* proliferating stem cell, *TA* transient amplifying, *TD* terminally differentiated; and, or – logical operators, *Q1* the lowest quartile of expression, *Q4* the highest quartile of expression

**Fig. 8:**
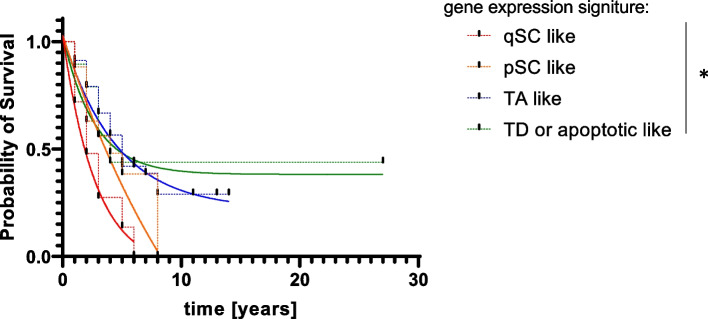
Survival analysis of patents with metastatic breast cancer stratified by genetic signature in their metastases. *- significant differences between curves, see text for details

Stratifying patients according to gene signatures that mimic tissue compartment was found to be more accurate and parsimonious model then the one which ignores such information (ΔAIC = 39.46, ER > 10^4^, *p* < 0.0001—extra sum of squares F test, *p* = 0.0007—log rank test) (Fig. 8). Furthermore, all the patients whose metastases were characterized by qSC signature (survival plateau or tail = 0 ± 6%) or pSC signature (survival plateau = 0 ± ≈0%) eventually died. However, patients with qSC signature (half-life = 1.9 ± 0.5 years) were dying almost 5 times faster than those with pSC signature (half-life = 9 ± 7.5 years) (η^2^ = 18.42%, ΔAIC = 2.96, ER = 4.4, *p* = 0.0111, Welch’s t test). Patients whose metastases expressed TA or TD like signatures exhibited survival plateaus that were well above 0%. More detailly, patients with TA signature plateaued at 22.2 ± 4.7% of survival probability and patients with TD signature had a survival probability tail at 37.4 ± 4.5%. If survival plateaus from all gene signatures are ordered in a manner that fallows usual tissue differentiation process (i.e. starting with qSC to pSC to TA and ending with TD) a rising exponential trend can be noticed (*R*^2^ = 7%, ΔAIC = 9.79, ER = 134.1, *p* = 0.0006).

## Discussion

We speculated that purpose of metastatic process might be providing an additional stroma which might be able to differentiate or induce quiescence in incoming metastatic cells. Additionally, we provided an artificial stroma in form of an implant in hope that this will augment differentiation or quiescence processes. Mice that went through a primary tumor removal and simultaneous implantation of ε-PCL implants without VEGF showed a relative decrease of lung metastatic load by 30% when compared to mice that had primary tumor removal only. The decrease in lung metastatic load was associated with an increase in proportion of tumor cells in the implant.

### The effect of ε-PCL implant without VEGF on metastatic burden

The ability of ε-PCL implants to attract breast cancer cells and decrease metastatic load to other organs was previously demonstrated by Azarin et al. [45]. Furthermore, in another study, the same group demonstrated that mice with the implants survive for longer period [46]. When compared to these two seminal studies, our study differs from the former in terms of primary tumor excision (i.e., Azarin et al. did not remove primary tumor) and from the latter in terms of tumor and ε-PCL scaffold implantation times (i.e., Rao et al. implanted the scaffolds 30 days before tumor implantation and excised the primary tumor after 10 days of growth). Our study adds to these in terms of translational potential because here we have shown explicitly that residual metastases after the removal of primary tumor can migrate to ε-PCL implant. This kind of experimental design mimics the clinical settings more faithfully and suggests that the implants might have a potential in treatment of residual metastatic disease. When it comes to survival of mice in our study, unfortunately, the Animal Welfare Committee did not give us the permission for the survival study, so we had to use the tumor load as the main outcome measure. If other types of cancer and their rodent models are considered, a study of exosome enriched intraperitoneal PCL implants in murine ovarian cancer (i.e., peritoneal carcinosis) model by de la Fuente et al. stands out as the one which demonstrated the greatest survival benefit [47]. Moreover, if the implants were removed after some time the survival benefit was even more dramatic. However, the mechanisms of these beneficial effects on tumor load and survival, they remain largely unknown.

### On the effects of metastatic process and ε-PCL implants on tumor cell differentiation

If the cancer lethality is considered from general systems theory point of view a following conjecture can be made: since the cause of tumor lethality is either an acute local complication (e.g., stenosis of hallow organ lumen, invasion of a blood vessel and thrombosis or simply destruction of organ parenchyma) or chronic systemic complication (i.e., cancer cachexia) [48], physiologically, it would make sense to postpone acute lethal complication by trading off local tumor volume for the equal cumulative volume that is dispersed in various organs, thus postponing the system failure. Moreover, since several studies have shown so far that normal tissue can induce quiescence or differentiation of invading cancer cells [11, 49], and thus stop or slow down cancer growth by normalizing tissue kinetics (i.e. the net difference between cell gain and loss), we have chosen to study the effects of the implants on differentiation and quiescence of metastatic cells.

We analyzed differentiation by quantifying proportions of different tissue compartments, through which cells pass during the maturational process. aCasp3, along with the Ki—67, was used as differentiation marker. The use of activated caspase 3 for such purpose is quite unusual, but the body of evidence from previous research [6, 7, 34, 50] and correlative analysis performed here, in our belief, can provide justification for such usage of aCasp3. We have shown that metastases, when compared to primary tumors, are associated with a shift from proliferating stem cell like (pSC) population to transient amplifying (TA) like cell population. The implants made such effect more uniform and complete. If expression signatures of tissue compartments in human metastases are taken into consideration, than this shift suggests a possible survival benefit. On the other hand, if the evidence that aCasp3 is a part of differentiation and that apoptosis is the ultimate step in differentiation or differentiation taken to extreme is disregarded, a different interpretation of flow cytometry results can be formulated. In this case, a metastatic process would be associated with an increase in aCasp3 expression, and the addition of implants would make such effect more complete and uniform. Increased expression of aCasp3 has been associated with sensitivity to chemotherapy and slower cell growth in breast cancer and this could also explain the differences in survival observed on human data [51, 52]. Thus, two different interpretations of data lead to the same conclusion.

When it comes to terminally differentiated or apoptotic like (TD) cells we can notice that both metastatic process and implants did not increase their proportions, therefore the effects of these processes on differentiation are only partial when it comes to differentiation, i.e., they only manage to differentiate cells to TA stage. Potential consequences of partial differentiation are twofold. Firstly, TA cells are also proliferating cells and therefore they increase the tumor load of an organism and eventually can contribute to lethal outcome. Secondly, due to the increased levels of aCasp3 this population should be more chemo or hormonal sensitive and slow growing then the stem cell populations and therefore in a setting of chemo or hormonal therapy, such as in metastatic breast cancer, might be the cause of prolonged survival.

### The quiescent tumor cells and VEGF enrichment of ε-PCL implant

If quiescent stem cell like (qSC) cells and VGEF enriched implants are considered the data are more puzzling. These cells were the most abundant in primary tumors, whereas mice treated with non-enriched implants had levels that were below detection threshold on flow cytometry. A group of mice treated with tumor excision only and the group that was treated with VEGF enriched implants, were not uniform when it comes to qSC cells. Roughly speaking, maximal proportions of qSC cells in these group were half the size of the maximal proportion of qSC cells in primary tumors. qSC tissue compartment signature was associated with the worst survival in metastatic breast patient’s cohort. This is not surprising since the mainstay therapy of patients with metastatic breast cancer is chemo and/or hormonal therapy [53]. Quiescent tumor cells, as any other quiescent tissue stem cells, do not proliferate nor do they express estrogen receptors [37]. However, they express proteins that are associated with the resistance to chemical toxicity such as ALDH, or ABC family transporters (which are often used as stemness markers) [54]. These properties make them quite resistant to any conventional chemo or hormonal therapy. The physiological role of quiescent stem cells is to proliferate and repopulate the stroma after tissue injury [3]. Thus, after the last cycle of chemotherapy quiescent tumor stem cells will simply repopulate the stroma and these cells will often be resistant to chemotherapeutic agents due to adaptation trough mutational changes or epigenetic modification in protein expression [55–57].

On the other hand, long term survival is sometimes associated with quiescent or dormant tumor metastases [58]. This observation can quite naturally be understood in terms of tumor load. The quiescence in tumor cells means that lethal tumor loads may be reached only after extended period of time, if ever. So far, it has been shown that tumor dormancy is induced by paracrine factors from surrounding stroma and differentiated cells of parenchyma [59, 60]. This may explain higher proportions of qSC cells in groups which were treated with primary tumor removal only and VEGF-enriched implant. Because these two groups had higher lung metastatic loads, they also had a higher absolute numbers of TA like cells. The latter are at least partially differentiated and thus could by paracrine means induce quiescence in proliferating stem cells.

The purpose of implanting VEGF-enriched implants was to establish a link between circulatory system and the implant sooner, so that more metastases might be attracted in the implant and thus an effect of tumor load reduction in lungs maximized. However, it turned out that the effect was the opposite, i.e., mice with VEGF-enriched implants had the greatest metastatic load in lungs. One reason for this might be a leak of loosely bound VEGF from the implant, although this is unlikely because the implants were washed in PBS for 24 h before implantation. Another reason might be a gradual release of bounded VEGF from the implant since ε-PCL is biodegradable material. Third and more complex reason might lie in a fact that ε-PCL once implanted gets infiltrated with various cells of myeloid origin, what could be more completely described as granulation or wound healing tissue [46]. This kind of tissue is known to mobilize mesenchymal stromal cells (MSC) from bone marrow by endocrine stimuli. MSCs, in turn, could cause at least partial differentiation or induce quiescence (i.e. lower proportion of pSC like compartment) of metastases in lungs [61]. The differentiation of epithelial cells is characterized by the loss of migration ability [62] and in this way the metastatic cells would simply get stuck in the lungs. The loss of migration ability would also explain the observation that VEGF-enriched implants were less infiltrated by tumor cells.

### Limitations and future experimentation

When it comes to limitations of the study two issues come to mind. The first one is marker or markers of cell differentiation and the way how different tissue compartments were defined in flow cytometric analysis. The former has been addressed earlier in the Results and Discussion sections, regarding the latter we used simple logic that tumor cells which do not express differentiation marker (aCasp 3) were considered stem cells, that could be further divided based on proliferation marker expression. Following the same logic cells that express differentiation marker and proliferate were considered as transient amplifying cells. Finally, tumor cells that were not proliferating and were expressing aCasp3 were either considered terminally differentiated or apoptotic.

The second issue is a 10 days of time lag between the primary tumor excision and the harvest of metastatic tissue in lungs. This means that, in a strict sense, the differences between these two tissues must be interpreted as effects of time, metastatic process and the implants. In alternative design we could have made an excision of primary tumor and harvest of metastatic tissue in a synchronous manner, however in this case it would be impossible to study the effect of the implants on metastases since, the implants need time to interact with metastases. The question of whether the differences between primary tumors and lung metastases in terms of differentiation can be attributed to the time and not to different biology (i.e. microenvironment) of primary and metastatic tumors, seems to be resolved in favor of microenvironment given the overwhelming evidence in literature [63].

When it comes to VEGF enrichment of the implants, this study would definitely benefit if dose response relationship between different VEGF concentrations and effects of implants on metastases could be elucidated.

Finally, when thinking of the future experimentation, given what has already been said in previous subsections, a next logical step is to induce complete instead of only partial differentiation in residual metastases. Theoretically, this might establish nongrowing tissue kinetics (i.e., rate of cell birth and apoptosis are at least equal) in leftover cancer. Off note, Fig. S3 might be instructive in this sense; it clearly visualizes how much tissues kinetics of lung metastases in mice treated with the implants is close to the normal respiratory epithelium kinetics. Mesenchymal stromal cells seem to be natural choice when considering the augmentation of the implant’s effects, since MSCs have so far shown capabilities to differentiate neoplastic cells. Furthermore, increasing the cumulative volume of the implants or removing the implant with replacement, as it was done by de la Fuente et al., also seems quite rational. Using the implants as chemo-sensitizing agent is also an option since transient amplifying cells should be more chemo-sensitive then quiescent or proliferating stem cells. However, this course of treatment seems riskier because the surviving quiescent stem cells might simply restore the cancerous tissue after the last dose of chemotherapy. This also points to the hypothesis that it might be useful to apply some kind of differentiation or quiescence inducing therapy (e.g., PGE_2_ [55] or iL6 antagonists, β blockers [60]) after the chemotherapy.

## Conclusion

ε-PCL implants without VEGF can reduce metastatic loads in lungs. The metastatic process reduces the average fraction of pSC and qSC like cells when compared to primary tumor. This effect is made more uniform by both types of ε-PCL implants.

## Supplementary Information

**Additional file 1.**

**Additional file 2.**

**Additional file 3.**

**Additional file 4.**

## Data Availability

Raw data is publicly available at FLOW Repository. http://flowrepository.org/public_experiment_representations
