# Supplementary material for: Eta polycaprolactone (ε-PCL) implants appear to cause a partial differentiation of breast cancer lung metastasis in a murine model
Source: BMC Cancer. 2023 Apr 13;23:343. doi: 10.1186/s12885-023-10813-6 (PMC10103376; doi:10.1186/s12885-023-10813-6)
Supplement: Supplementary file 1 — Additional file 1. [file 12885_2023_10813_MOESM1_ESM.pptx]

## Slide 1
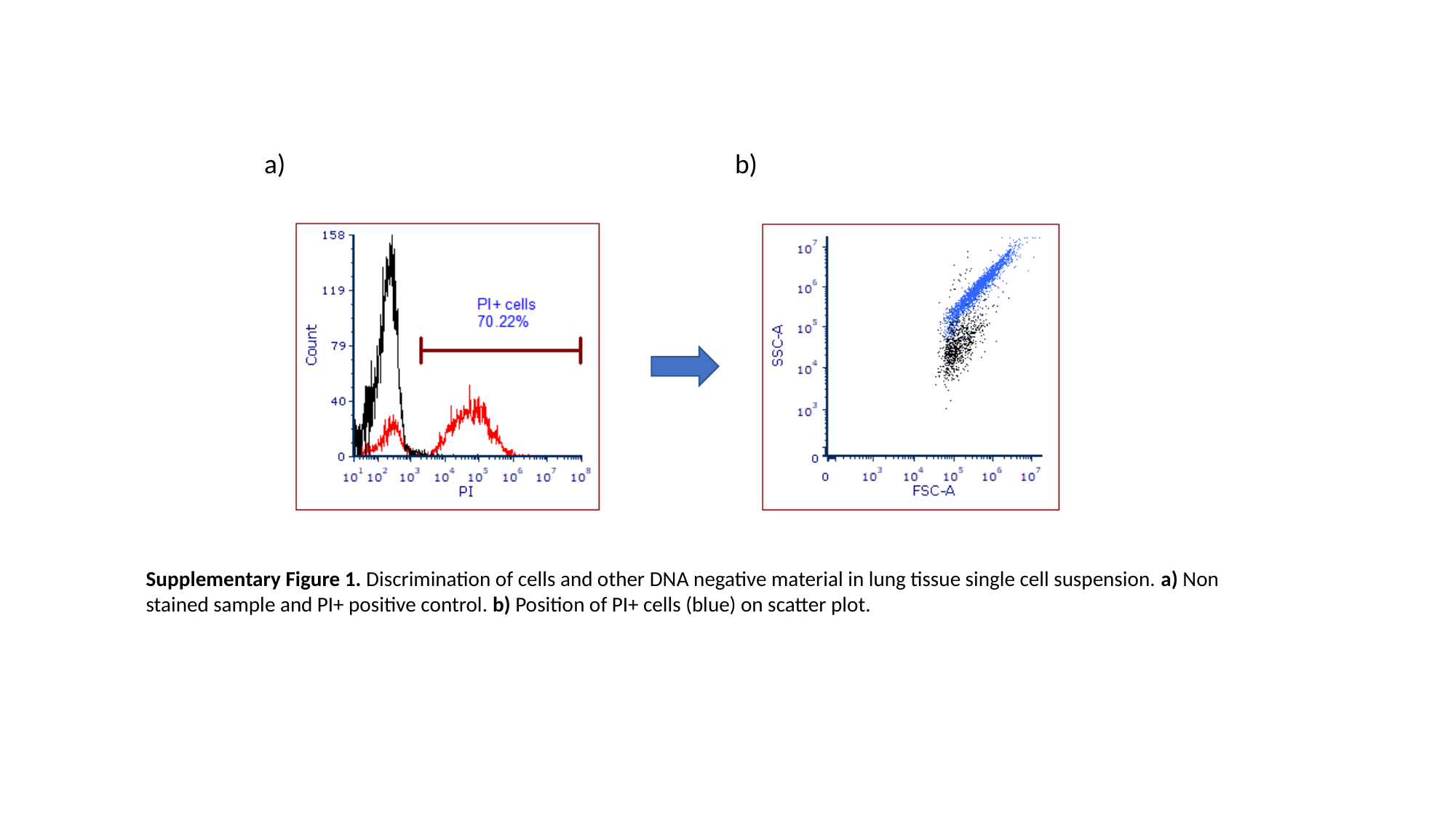

a)
b)
Supplementary Figure 1. Discrimination of cells and other DNA negative material in lung tissue single cell suspension. a) Non stained sample and PI+ positive control. b) Position of PI+ cells (blue) on scatter plot.

## Slide 2
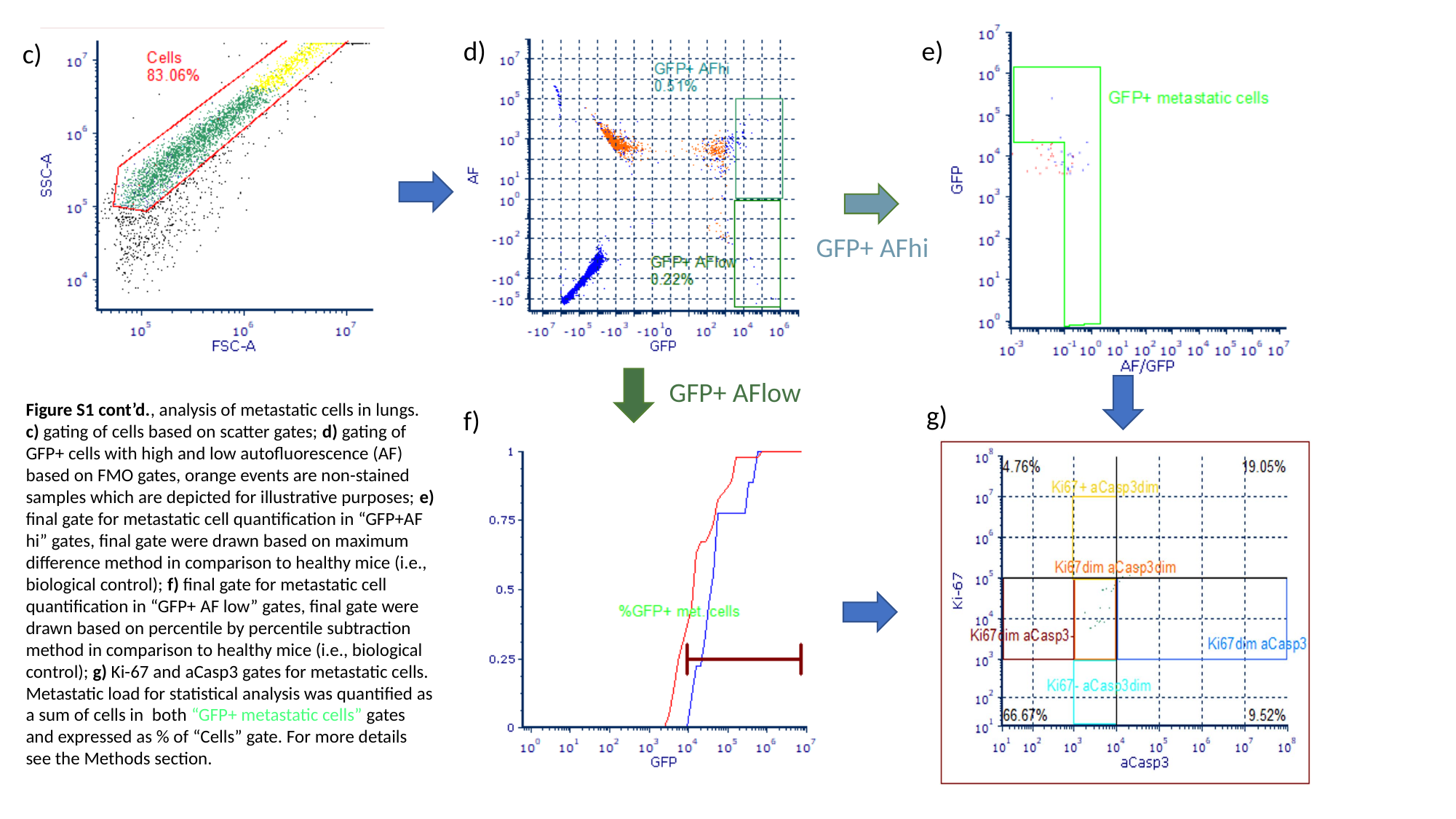

d)
e)
c)
GFP+ AFhi
GFP+ AFlow
Figure S1 cont’d., analysis of metastatic cells in lungs. c) gating of cells based on scatter gates; d) gating of GFP+ cells with high and low autofluorescence (AF) based on FMO gates, orange events are non-stained samples which are depicted for illustrative purposes; e) final gate for metastatic cell quantification in “GFP+AF hi” gates, final gate were drawn based on maximum difference method in comparison to healthy mice (i.e., biological control); f) final gate for metastatic cell quantification in “GFP+ AF low” gates, final gate were drawn based on percentile by percentile subtraction method in comparison to healthy mice (i.e., biological control); g) Ki-67 and aCasp3 gates for metastatic cells. Metastatic load for statistical analysis was quantified as a sum of cells in both “GFP+ metastatic cells” gates and expressed as % of “Cells” gate. For more details see the Methods section.
g)
f)

## Slide 3
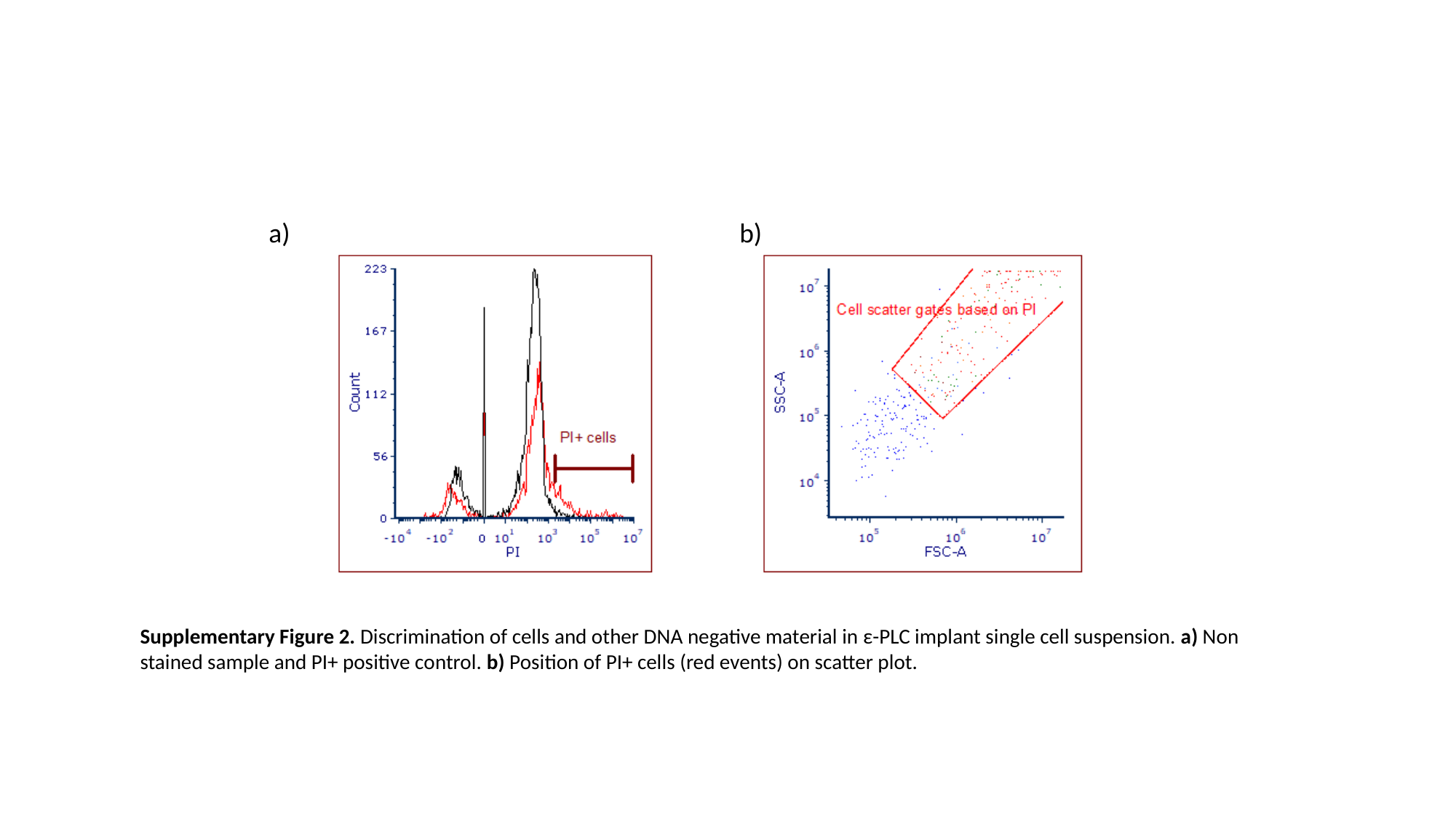

a)
b)
Supplementary Figure 2. Discrimination of cells and other DNA negative material in ε-PLC implant single cell suspension. a) Non stained sample and PI+ positive control. b) Position of PI+ cells (red events) on scatter plot.

## Slide 4
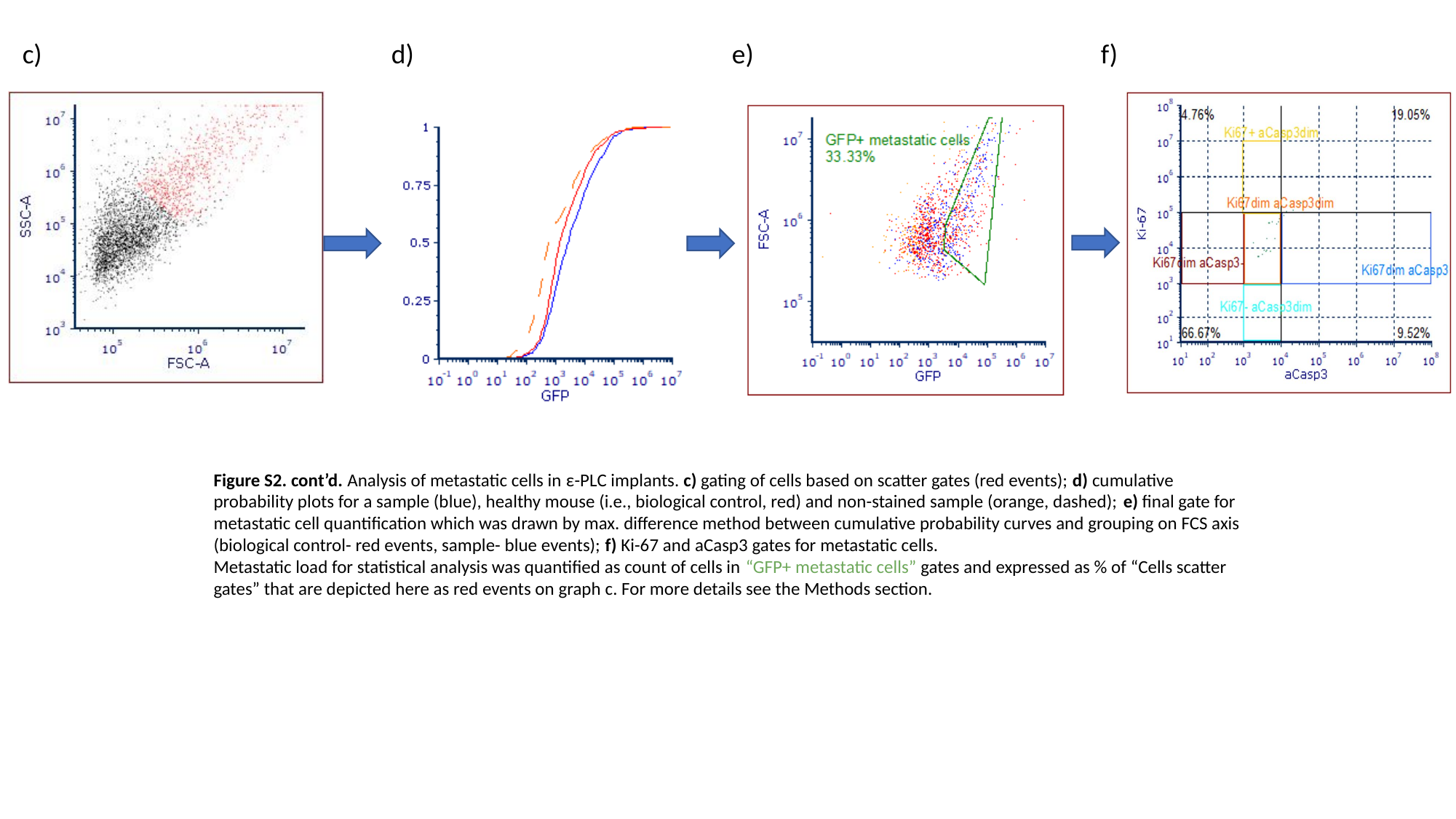

c)
e)
f)
d)
Figure S2. cont’d. Analysis of metastatic cells in ε-PLC implants. c) gating of cells based on scatter gates (red events); d) cumulative probability plots for a sample (blue), healthy mouse (i.e., biological control, red) and non-stained sample (orange, dashed); e) final gate for metastatic cell quantification which was drawn by max. difference method between cumulative probability curves and grouping on FCS axis (biological control- red events, sample- blue events); f) Ki-67 and aCasp3 gates for metastatic cells.
Metastatic load for statistical analysis was quantified as count of cells in “GFP+ metastatic cells” gates and expressed as % of “Cells scatter gates” that are depicted here as red events on graph c. For more details see the Methods section.
