# Supplementary material for: Eta polycaprolactone (ε-PCL) implants appear to cause a partial differentiation of breast cancer lung metastasis in a murine model
Source: BMC Cancer. 2023 Apr 13;23:343. doi: 10.1186/s12885-023-10813-6 (PMC10103376; doi:10.1186/s12885-023-10813-6)
Supplement: Supplementary file 2 — Additional file 2. [file 12885_2023_10813_MOESM2_ESM.pptx]

## Slide 1
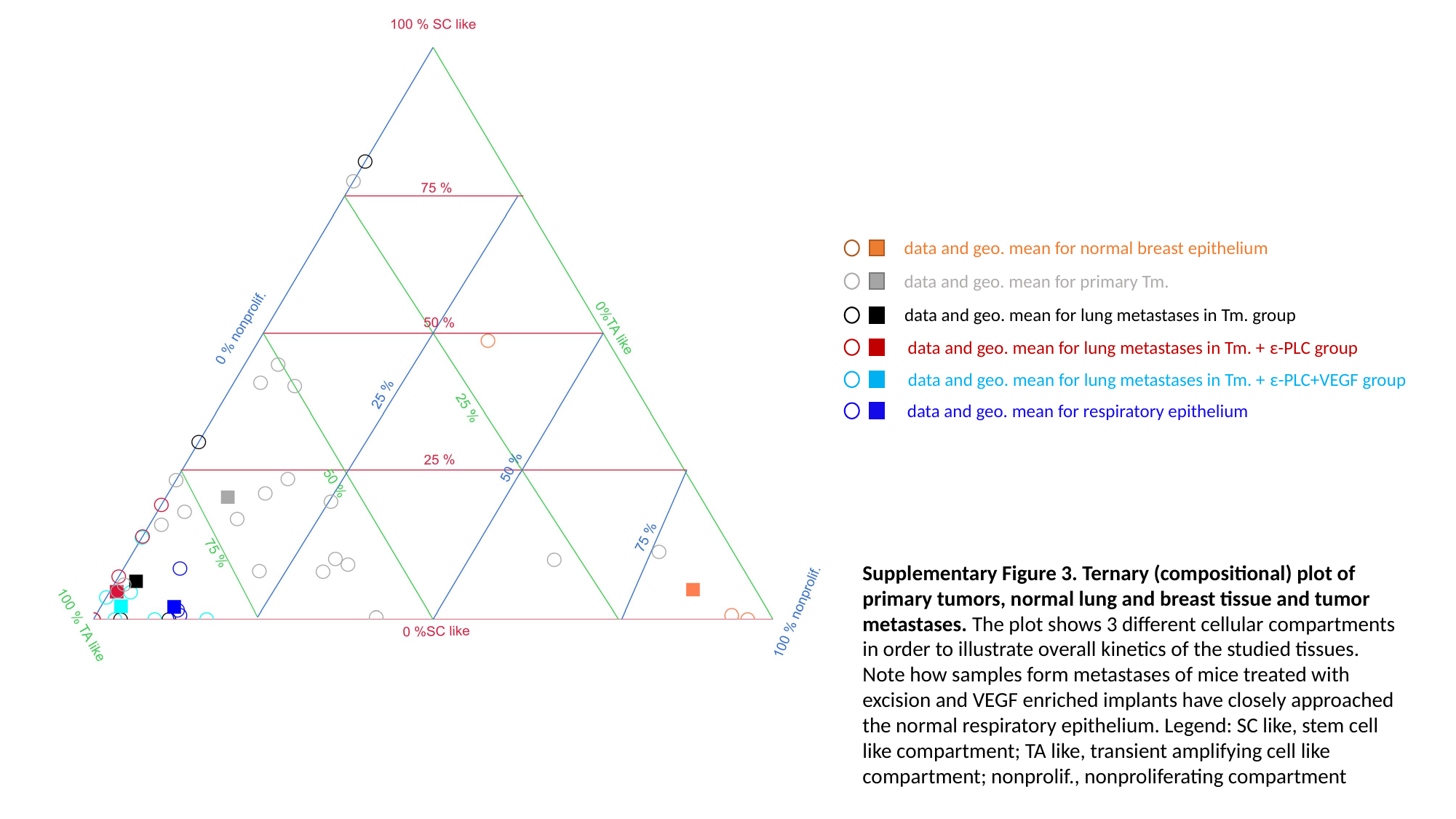

data and geo. mean for normal breast epithelium
data and geo. mean for primary Tm.
data and geo. mean for lung metastases in Tm. group
data and geo. mean for lung metastases in Tm. + ε-PLC group
data and geo. mean for lung metastases in Tm. + ε-PLC+VEGF group
data and geo. mean for respiratory epithelium
Supplementary Figure 3. Ternary (compositional) plot of primary tumors, normal lung and breast tissue and tumor metastases. The plot shows 3 different cellular compartments in order to illustrate overall kinetics of the studied tissues. Note how samples form metastases of mice treated with excision and VEGF enriched implants have closely approached the normal respiratory epithelium. Legend: SC like, stem cell like compartment; TA like, transient amplifying cell like compartment; nonprolif., nonproliferating compartment
