# Supplementary material for: Eta polycaprolactone (ε-PCL) implants appear to cause a partial differentiation of breast cancer lung metastasis in a murine model
Source: BMC Cancer. 2023 Apr 13;23:343. doi: 10.1186/s12885-023-10813-6 (PMC10103376; doi:10.1186/s12885-023-10813-6)
Supplement: Supplementary file 3 — Additional file 3. [file 12885_2023_10813_MOESM3_ESM.docx]

**Table S1.** Correlation between caspase 3 and markers of stemness or differentiation during secretory differentiation and peak differentiation phases of breast growth and differentiation in pregnant mice

| Stem cell markers | Coefficient of correlation (r)* | P value* |
| --- | --- | --- |
| *ALDH1* | -0.56 | <0.0001 |
| *CK19* | -0.57 | <0.0001 |
| *CD24* | -0.23 | 0.0003 |
| *CD29* | -0.33 | 0.0002 |
| *CD49f* | -0.09 | 0.0004 |
| *nestin* | -0.12 | 0.0004 |
| Morphogens |  |  |
| *Epimorphin* | -0.49 | 0.0001 |
| Markers of differentiation |  |  |
| *PRLR* | 0.75 | <0.0001 |
| *SREBP* | 0.62 | <0.0001 |
| *STAT5* | 0.37 | 0.0002 |
| *WAP* | 0.60† | 0.013† |
| *all caseins* | 0.46 | 0.0001 |
| *α- lactalbumin* | 0.84 | <0.0001 |
| *GLUT - 1* | 0.71 | 0.0003 |
| *Glycam 1* | 0.38 | 0.0002 |
| *CK6* | 0.76 | <0.0001 |

*Correlations were calculated using linear model, with exception of WAP, and reduced mean axis (RMA) fitting procedure.

†Quadratic model with OLS fit.
