# Supplementary material for: Eta polycaprolactone (ε-PCL) implants appear to cause a partial differentiation of breast cancer lung metastasis in a murine model
Source: BMC Cancer. 2023 Apr 13;23:343. doi: 10.1186/s12885-023-10813-6 (PMC10103376; doi:10.1186/s12885-023-10813-6)
Supplement: Supplementary file 4 — Additional file 4. [file 12885_2023_10813_MOESM4_ESM.docx]

Table S2. Distributions used for modelling of quiescent stem (qSC) cell like population among GFP+ cells in the lungs

| Experimental group | Distribution | Location parameter (μ) | Scale parameter (β) | x_max_ | Arithmetic mean‡ | Mode‡ | 0.5% trimmed range‡ | R^2^ |
| --- | --- | --- | --- | --- | --- | --- | --- | --- |
| primary tumor (pTm) | exponential* | -0.6251 ± 0.31 | 6.235 ± 0.55 | NA | 5.64 ± 0.85 % | 0 ± ≈0 % | 0 to 32.4 % | 95% |
| pTm. excision | power law† | -0.2192 ± 0.27 | -0.7325 ± 0.05 | 11.17 ± 0.71 % | Not defined | 0 ± 0.05 % | 0 to 10.95 % | 99% |
| pTm. Excision + ε – PLC implant | all datapoints equal 0 |  |  |  |  |  |  |  |
| pTm. Excision + ε – PLC implant + VEGF | power law† | -2.958 ± 0.69 | -0.7108 ± 0.03 | 17.54 ± 1.07 % | Not defined | 0 ± ≈0 % | 0 to 17.18 % | 99% |

Legend: * $cdf=1-e^{\frac{\mu-x}{\beta}}arithmetic mean = \beta+\mu$ , , *mode* = *μ*.

† $cdf=({\frac{x-\mu}{x_{max}-\mu})}^{(\beta+1)}$, arithmetic mean is not defined if β < 0, mode = *μ*; x_max_ – maximum value of dependent variable.

‡ if an estimate is negative percentage or greater than 100% , than the estimate is rounded off to 0 % or 100 %, respectively;

Interval estimate is given as standard error, i.e., 68% CI

Table S3. Distributions used for modelling of proliferating stem (qSC) cell like population among GFP+ cells in the lungs

| Experimental group | Distribution | Location parameter (μ) | Scale parameter (β) | x_max_ | Arithmetic mean† | Mode† | 0.5% trimmed range† | R^2^ |
| --- | --- | --- | --- | --- | --- | --- | --- | --- |
| primary tumor (pTm) | lognormal* | 1.274 ± 0.02* | 0.3621 ± 0.01* | NA | 22 ± 1.5 % | 13.89 ± 0.3 % | 2.19 to 100 % | 77% |
| pTm. excision | power law‡ | -1.597 ± 2.13 | -0.7241 ± 0.05 | 80.54 ± 5 % | not defined | 0 ± 0.53 % | 0 to 79.06 % | 99% |
| pTm. Excision + ε – PLC implant | power law | -0.8287 ± 1.2 | -0.3184 ± 0.16 | 20.7 ± 1.7 % | not defined | 0 ± 1.2 % | 0 to 20.54 % | 97% |
| pTm. Excision + ε – PLC implant + VEGF | power law | -0.1318 ± 0.37 | -0.7521 ± 0.02 | 14.35 ± 0.54 | not defined | -0.1318 ± 0.37 % | 0 to 14.05 % | 99% |

Legend: * normal (Gaussian) distribution was fitted to log_10_ transformed data, $arithmetic mean ={10}^{\mu+\frac{\beta^{2}}{2}}$ , $mode={10}^{\mu-\beta^{2}}$;

† The estimates are given on original scale and if an estimate is negative percentage or greater than 100%, than the estimate is rounded off to 0 % or 100 %, respectively.

‡ $cdf=({\frac{x-\mu}{x_{max}-\mu})}^{(\beta+1)}$, arithmetic mean is not defined if β < 0, mode = *μ*, x_max_ – maximum value of dependent variable.

Interval estimate is given as standard error, i.e., 68% CI

Table S4. Distributions used for modelling of transient amplifying like cells (TA) population among GFP+ cells in the lungs

| Experimental group | Distribution | Location parameter (μ) | Scale parameter (β) | x_max_ | Arithmetic mean‡ | Mode‡ | 0.5% trimmed range‡ | R^2^ |
| --- | --- | --- | --- | --- | --- | --- | --- | --- |
| primary tumor (pTm) | Gaussian | 60.77 ± 0.7 | 16.50 ± 2 | NA | 60.77 ± 0.7 % | 60.77 ± 0.7 % | 18.26 to 100 % | 97% |
| pTm. excision | exponential* | 0.009 ± 0.0003 | 0.002 ± 0.0005 | NA | 100 ± 0 % | 100 ± ≈0% | 0.99 to 100 % | 97% |
| pTm. Excision + ε – PLC implant | power law† | 0.008 ± 0.0003 | -0.02 ± 0.14 | 0.01251 ± ≈ 0% | Not defined | 100 ± ≈0% | 80.07 to 100 % | 99% |
| pTm. Excision + ε – PLC implant + VEGF | uniform | 81.93§ | 100.6§ | NA | 91.26± 0.93 % | Not defined | 81.93 to 100 % | 94% |

Legend: * Exponential distribution ($cdf=1-e^{\frac{\mu-x}{\beta}}$) was fitted to inversely transformed (1/x) data, $arithmetic mean = \beta+\mu$, *mode* = *μ*.

† Power law distribution ($cdf=({\frac{x-\mu}{x_{max}-\mu})}^{(\beta+1)}$) was fitted to inversely transformed (1/x) data, arithmetic mean is not defined if β < 0, mode = *μ*, x_max_ – maximum value of dependent variable.

‡ The estimates are given on original scale and if an estimate is negative percentage or greater than 100%, than the estimate is rounded off to 0 % or to a 100 %, respectively.

§ Values are minimum and maximum since uniform distribution does not have a scale and location parameter.

Interval estimate is given as standard error, i.e., 68% CI.

Table S5. Distributions used for modelling terminally differentiated or apoptotic (TD) like cells population among GFP+ cells in the lungs

| Experimental group | Distribution | Location parameter (μ) | Scale parameter (β) | x_max_ | Arithmetic mean‡ | Mode‡ | 0.5% trimmed range‡ | R^2^ |
| --- | --- | --- | --- | --- | --- | --- | --- | --- |
| primary tumor (pTm) | exponential* | -5.849 ± 1.07 | 11.92 ± 1.35 | NA | 6.07 ± 2.5 % | 0 ± ≈0 % | 0 to 57.31 % | 95% |
| pTm. excision | all datapoints equal 0 |  |  |  |  |  |  |  |
| pTm. Excision + ε – PLC implant | all datapoints equal 0 |  |  |  |  |  |  |  |
| pTm. Excision + ε – PLC implant + VEGF | triangular† | 0.81 ± ≈0† | 3.79 ± ≈0† | NA | 1.81 ± ≈0 | 0.81 ± ≈0 | 0.81 to 3.57 % | ≈100% |

Legend: * $cdf=1-e^{\frac{\mu-x}{\beta}}arithmetic mean = \beta+\mu$ , *mode* = *μ*.

† $pdf=\left\{ \begin{aligned} \frac{2(c-x)}{{(c-a)}^{2}},x>a \\ 0, x\leq a \end{aligned} \right.$ usually, triangular distribution pdf has 3 parameters (*a* for minimum, *b* for mode, and *c* for maximum), here we used a simplification in which *a=b*. Furthermore, *a* is given in column with heading Location parameter (μ) and *c* is given in column with heading Scale parameter (β). $arithemtic mean=\frac{a+b+c}{3}$, $mode=a=b$

‡ If an estimate is negative percentage or greater than 100%, than the estimate is rounded off to 0 % or 100 %, respectively.
